# Exploring European Consensus About the Remaining Treatment Challenges and Subsequent Opportunities to Improve the Management of Invasive Fungal Infection (IFI) in the Intensive Care Unit

**DOI:** 10.1007/s11046-024-00852-3

**Published:** 2024-05-05

**Authors:** Martin Hoenigl, David A. Enoch, Dominic Wichmann, Duncan Wyncoll, Andrea Cortegiani

**Affiliations:** 1 Division of Infectious Diseases, Medical University of Graz, Auenbruggerplatz 15, 8036 Graz, Austria; 2 BioTechMed-Graz, Graz, Austria; 3 ECMM Excellence Center for Medical Mycology, Translational Medical Mycology Research Unit, Medical University of Graz, Graz, Austria; 4 Clinical Microbiology & Public Health Laboratory, UK Health Security Agency, Cambridge University Hospital NHS Foundation Trust, Addenbrookes Hospital, Cambridge, UK; 5 Department of Intensive Care Medicine, University Medical Center of Hamburg-Eppendorf, Hamburg, Germany; 6 Department of Intensive Care, Guy’s and St Thomas’ Hospital, London, UK; 7 Department of Precision Medicine in Medical, Surgical and Critical Care (Me.Pre.C.C.), University of Palermo, Palermo, Italy; 8 Department of Anesthesia Intensive Care and Emergency, University Hospital Policlinico ‘Paolo Giaccone, Palermo, Italy

**Keywords:** Delphi study, Candidaemia, Aspergillosis, Invasive fungal infections, Intensive care units

## Abstract

**Background:**

The global prevalence of invasive fungal infections (IFI) is increasing, particularly within Intensive Care Units (ICU), where *Candida* spp. and *Aspergillus* spp. represent the most important pathogens. Diagnosis and management of IFIs becomes progressively challenging, with increasing antifungal resistance and the emergence of rare fungal species.

Through a consensus survey focused on assessing current views on how IFI should be managed, the aim of this project was to identify challenges around diagnosing and managing IFIs in the ICU. The current status in different countries and perceived challenges to date amongst a multidisciplinary cohort of healthcare professionals involved in the care of IFI in the ICU was assessed.

**Methods:**

Using a modified Delphi approach, an expert panel developed 44 Likert-scale statements across 6 key domains concerning patient screening and minimal standards for diagnosis of IFIs in ICU; initiation and termination of antifungal treatments and how to minimise their side effects and insights for future research on this topic. These were used to develop an online survey which was distributed on a convenience sampling basis utilising the subscriber list held by an independent provider (M3 Global). This survey was distributed to intensivists, infectious disease specialists, microbiologists and antimicrobial/ICU pharmacists within the UK, Germany, Spain, France and Italy. The threshold for consensus was set at 75%.

**Results:**

A total of 335 responses were received during the five-month collection period. From these, 29/44 (66%) statements attained very high agreement (≥ 90%), 11/44 (25%) high agreement (< 90% and ≥ 75%), and 4/44 (9%) did not meet threshold for consensus (< 75%).

**Conclusion:**

The results outline the need for physicians to be aware of the local incidence of IFI and the associated rate of azole resistance in their ICUs. Where high clinical suspicion exists, treatment should start immediately and prior to receiving the results from any diagnostic test. Beta-D-glucan testing should be available to all ICU centres, with results available within 48 h to inform the cessation of empirical antifungal therapy. These consensus statements and proposed measures may guide future areas for further research to optimise the management of IFIs in the ICU.

**Supplementary Information:**

The online version contains supplementary material available at 10.1007/s11046-024-00852-3.

## Background

In recent years the global prevalence of invasive fungal infections (IFIs) has increased across many clinical environments but particularly within intensive care units (ICU). [1–3] For instance, over the past decade the rate of candidaemia has increased in some settings by up to 50% [2] with an incidence of 5.1/1000 ICU admissions. [3]

Infections from *Candida* species and invasive aspergillosis (IA) represent a critical issue for patients within an ICU. *Candida* species are responsible for approximately 80% of IFI infections [4, 5] and candidaemia, the most common clinical presentation of invasive candidiasis, has a high mortality ranging from 28–59%. [6] Indeed, candidaemia accounted for 7.7% of cases in a large multicentre epidemiological study of hospital-acquired bloodstream infection in the ICU. [7] In recent years, infections by *Aspergillus* spp. in the form of IA have been frequently reported in critically ill patients, especially in patients with severe COVID-19, influenza, or chronic obstructive pulmonary disease (COPD), as well as the very old and critically ill. [8–11]

Patients admitted to an ICU with severe influenza or COVID-19 introduce a new set of challenges. These patients are at a high risk of developing an IFI due to factors such as epithelial damage caused by viral infection, mechanical ventilation, and immunosuppression. [12, 13] Aside from COVID-19 associated pulmonary aspergillosis (CAPA), COVID-19 associated mucormycosis (CAM), and *Pneumocystis* *jirovecii* pneumonia (PJP) (all of which increase patient mortality) [12, 14], infections with rare moulds and rare yeast have also been reported [12] increasing difficulties in patient management.

Depending on the presence of risk factors, underlying diseases and geographic location, IA may occur in 1 to 20% of ICU patients [4], and (in the absence of viral pandemics) approximately 43–80% of these do not have a haematological malignancy. [5] Due to the absence of specific clinical and radiological findings, diagnosis remains a challenge, as shown in a 2019 autopsy study where only 40% of IA cases were diagnosed in vivo [15]*.* In the absence of early diagnosis, mortality for IA cases is up to 80%. [6]

The epidemiology of IFI is changing as multi-resistant strains of not only *Candida* but also *Aspergillus* are emerging. [16, 17] The observed increase of non-albicans *Candida* spp., often showing resistance to azoles, is also a major concern for prolonged hospitalizations. [18–20] There is an increasing incidence of rare mould and yeast infections which have previously been geographically restricted. [17] This is thought to be caused by advances in diagnostics, allowing further differentiation and detection of previously unknown pathogens as well as broad usage of antifungals. Finally, the concern of climate change resulting in the emergence of new fungal pathogens that are adapting to human temperature, like *Candida auris* [1, 21]*.*

Being able to treat an IFI rapidly is important for survival, yet may be hampered by the complexity of obtaining an accurate diagnosis. [6, 22] Apart from candidaemia, tissue sampling with histological examination remains the gold standard but is often not possible while patients are still alive. In addition, sensitivity of culture is usually below 50%. [22] In the absence of a reliable gold standard, non-culture-based tests are increasingly used for diagnosis of IFI in the ICU. The most important tests utilized are beta-D-glucan (BDG) for invasive *Candida* infections (sensitivity 77%, specificity 85%) [23], and galactomannan (GM) from bronchioalveolar lavage fluid (BALF) for invasive aspergillosis including viral associated pulmonary aspergillosis (sensitivity 75–86%, specificity 94–95%). [24, 25] The availability of these tests varies across Europe, and the tests are suboptimal in terms of sensitivity and specificity. [1, 22] The patient population admitted to ICU is heterogenous, and infected individuals often present with non-specific signs, symptoms, and radiological findings. [22]

Building on previous work in this area [1, 26], the aim of this consensus survey was to: (i) gauge the views of a multidisciplinary cohort of healthcare professionals (HCPs) regarding the current clinical reality of management; (ii) describe what is the optimal care of IFIs in the ICU; and (iii) ultimately identify remaining challenges around diagnosing and managing IFIs in the ICU.

## Methods

A multidisciplinary panel (the study authors) from across Western Europe, selected based on their publications, leadership in international scientific societies, clinical expertise, and geographical representation of the countries where the survey was validated, convened in May 2022 to discuss remaining challenges in the management of IFIs in ICUs.

Using a modified Delphi methodology and a structured meeting format guided by an independent facilitator (Triducive Partners Limited, St Albans, UK), the expert panel identified 6 main domains of focus:

A) Increasing IFI suspicion (patient characteristics/co-morbidities) and screening
B) Accessibility and minimum standard for diagnosis
C) Initiation and change of treatment
D) Termination of treatment
E) Managing side effects and drug-drug interactions
F) Future guidance and data requirements

These domains were developed and discussed in detail by the expert panel during the initial project meeting and an initial set of statements were developed. The initial statements were then re-circulated amongst the members of the expert panel for individual ratification and amendment. A set of 44 statements were finally agreed for wider testing amongst ICU HCPs. These statements were used to form an online survey, which was distributed to ICU HCPs (intensivists, infectious disease specialists, microbiologists, and antimicrobial and/or ICU pharmacists) in five of the largest European markets (France, Germany, Italy, Spain and the UK) typically grouped as a source of data for European work.

The survey was shared on a convenience sampling basis by an independent company (M3 Global, London, UK) to their database of subscribers. Responses were sought using an incentivised methodology through standard market research approach. The invitation was unique and could not be redistributed. Each respondent was only able to complete the survey once. [27] Respondents were screened before completing the survey as to their level of experience within ICU departments and what familiarity they had with treating and diagnosing IFIs. To be eligible to take part in the survey, physicians would need a minimum of 2–5 years’ experience in their speciality as an intensivist or infectious diseases specialist, and a minimum of 5 years’ experience as a pharmacist or microbiologist.

Triducive Partners Limited independently managed meeting logistics, facilitation, structuring of information flow and survey management to ensure the anonymity of responders.

Stopping criteria were established as a minimum of 50 responses from each country surveyed, and a five-month window to collect responses (September 2022 to January 2023). A threshold for consensus was agreed at 75%. This was further defined as ‘high’ at ≥ 75% to < 90% and ‘very high’ at ≥ 90%.

The questionnaire was composed of each statement along with a 4-point Likert scale (‘strongly disagree’, ‘tend to disagree’, ‘tend to agree’, and ‘strongly agree’) to allow respondents to indicate their corresponding level of agreement. The questionnaire also captured some demographic data on country and role for further analysis. A statement of consent was included at the start of the survey, and consent was implied by completion and submission of the survey. As this study only sought the anonymous opinions of HCPs and no patient specific data was captured, ethical approval was deemed not required.

Completed surveys were anonymously collated and analysed by the independent facilitator to produce an arithmetic agreement score for each statement. The result was reviewed by the panel who subsequently proposed a set of measures aimed to address the challenges identified by the consensus survey.

## Results

Following from the initial meeting held in May 2022 an initial set of 37 statements was constructed for review by the expert panel. During the independent review and approval stages, this set was refined to a final approved set of 44 statements which was then developed into the survey used within this study (Table 1).

**Table 1:** Defined consensus statements distributed for testing and corresponding levels of agreement attained

No	Statement	Agreement (%)
*Topic A: Increasing invasive fungal infection suspicion (patient characteristics/co-morbidities) and screening*		
1	The risk of developing invasive fungal infection is dependent on patient characteristics	95
2	Every patient in the intensive care units (ICU) is at a risk of developing an invasive fungal infection	84
3	Risk factors should always be considered along with results from other diagnostic tools when considering treatments for invasive fungal infection	96
4	Physicians should be aware of the true incidence of invasive fungal infection in the ICU unit where they work	94
5	Intubated patients who show an unexplained deterioration of respiratory function during an ICU stay should be screened for aspergillosis	89
6	Patients where the suspicion for invasive pulmonary aspergillosis should be high include COPD patients, immunocompromised patients, patients with viral pneumonia (i.e. severe influenza, COVID-19), patients with liver cirrhosis, and patients with autoimmune diseases	96
7	Patients at a high risk of invasive *Candida* infections include those with any disruption of the skin and gastrointestinal barrier and those receiving treatments that change the composition of the human gut microbiome (e.g., patients with haematological problems, patients using IV drugs, patients on prolonged broad-spectrum antibiotics, cases of total parenteral nutrition, gastrointestinal surgery cases, patients with advanced liver cirrhosis, patients with severe sepsis, patients for hepatological surgery, patients with burns, patients requiring catheters)	95
8	Respiratory *aspergillosis* has a spectrum from airway colonisation to airway-invasion to angio-invasion. Once angio-invasion occurs, mortality is very high despite appropriate antifungal therapy	94
9	The established diagnostic criteria and tools for invasive aspergillosis in patients who are neutropenic often cannot be applied to patients who are non-neutropenic due to differences in patient characteristics between the two groups	86
*Topic B: Accessibility and minimum standard for diagnosis*		
10	Access to a Beta-D glucan fungal antigen result takes longer than 3 days in my institution	68
11	Access to a serum galactomannan antigen result takes longer than 3 days in my institution	61
12	Access to a serum Beta-D glucan fungal antigen result should be available within 48 h	96
13	Access to a BAL or serum galactomannan antigen result should be available within 48 h	96
14	Access to *Pneumocystis* *jirovecii* PCR should be available in all ICU units within 48 h	96
15	Access to fungal culture, species identification methods, and susceptibility testing should be available at every hospital	90
16	The ideal sample for *aspergillosis* in the ICU is a BAL for galactomannan and culture	93
*Topic C: Initiation and change of treatment*		
17	Where high clinical suspicion exists, treatment should start immediately and prior to receiving the results from any diagnostic test	94
18	Where resistance is known to be > 10% for azoles generally in the local centre, alternative choice of treatment should be considered	90
19	In cases where culture/specific diagnostics are not possible, a broad spectrum anti-fungal treatment should be chosen	91
20	Serum beta-D glucan antigen test should be taken before initiation of any anti-fungal treatment for patients suspected with *Candida* infection	77
21	Voriconazole is not a suitable treatment option for ICU patients	42
22	If voriconazole is used for treatment, access to therapeutic drug monitoring of voriconazole should be available within 48 h after achieving steady state to optimise the treatment regimen	91
23	If voriconazole is used for treatment, access to levels of voriconazole should be available within 24 h after achieving steady state to optimise the treatment regimen	84
24	If voriconazole fails to reach the required therapeutic levels after reaching steady state, either a dose escalation or a change to another antifungal would be the most appropriate strategy	92
25	The choice of treatment for an invasive fungal infection should also be based on pharmacokinetics and site of infection	94
26	The fungicidal mode of action of anti-fungal treatment in abdominal infections is an important treatment attribute	91
27	If susceptibility/MIC data are not available, choose antifungal treatment according to spectrum of activity and patient response	94
28	Data on antifungal treatment in patients on ECMO are limited and further studies are needed	89
29	Patients on ECMO have generally lower levels of antifungals, therefore either higher dosages or TDM with rapid dosage adjustments is needed	90
30	Azole resistance in aspergillus is an emerging problem that requires future action	87
*Topic D: Termination of treatment*		
31	If the Beta-D glucan result obtained before initiation of antifungal therapy is low (below 79 pg/mL), then treatment should be ceased when there is no other mycological evidence for an IFD	80
32	In treatment of suspected (and not proven) *Candida* infection without a clear clinical focus, if no improvement in clinical situation is seen within 4–5 days, then treatment can be stopped	69
*Topic E: Managing side effects and drug-drug interactions*		
33	Drug-drug interactions are an important consideration when selecting treatments for patients	97
34	Any antifungal treatment should be selected based on the ability in the hospital to measure the effects of drug interactions (such as drug-levels)	82
35	Co-morbidities are an important consideration when selecting treatments for patients	96
36	In patients with renal impairment, optimal choice of antifungal treatment needs to be weighed against the diagnosis of fungal Infection and renal replacement therapy options	96
*Topic F: Future guidance and data requirements*		
37	More observational data is required (specifically from the ICU) on the incidence of invasive fungal infection for specific/defined patient groups	94
38	The disparities of diagnosing patients with invasive fungal infections results in a lack of clarity around accurate incidence	91
39	Different therapeutic strategies for *Aspergillus* should be applied to different areas of incidence levels	91
40	Different therapeutic strategies for rare yeast and rare moulds should be applied to different areas of incidence levels	90
41	In areas of higher overall geographical incidence of *Aspergillus*, the role of prophylactic treatment should be considered	75
42	Randomised controlled trials are needed to guide the role of *Aspergillus* prophylaxis in the at-risk ICU population (i.e., where risk and prevalence is higher)	95
43	Proactive formal training is required to improve clinical confidence in using anti-fungal treatments and interpreting the diagnostic testing results	92
44	There is a general lack of confidence in using anti-fungal treatments and interpreting the diagnostic testing results	79

*BAL* Bronchoalveolar lavage, *COPD* Chronic obstructive pulmonary disease, *ECMO* Extracorporeal membrane oxygenation, *IFD* Invasive fungal disease, *IV* intravenous, *MIC* minimum inhibitory concentration, *PCR* Polymerase chain reaction, *TDM* Therapeutic drug monitoring

**Fig. 1:**
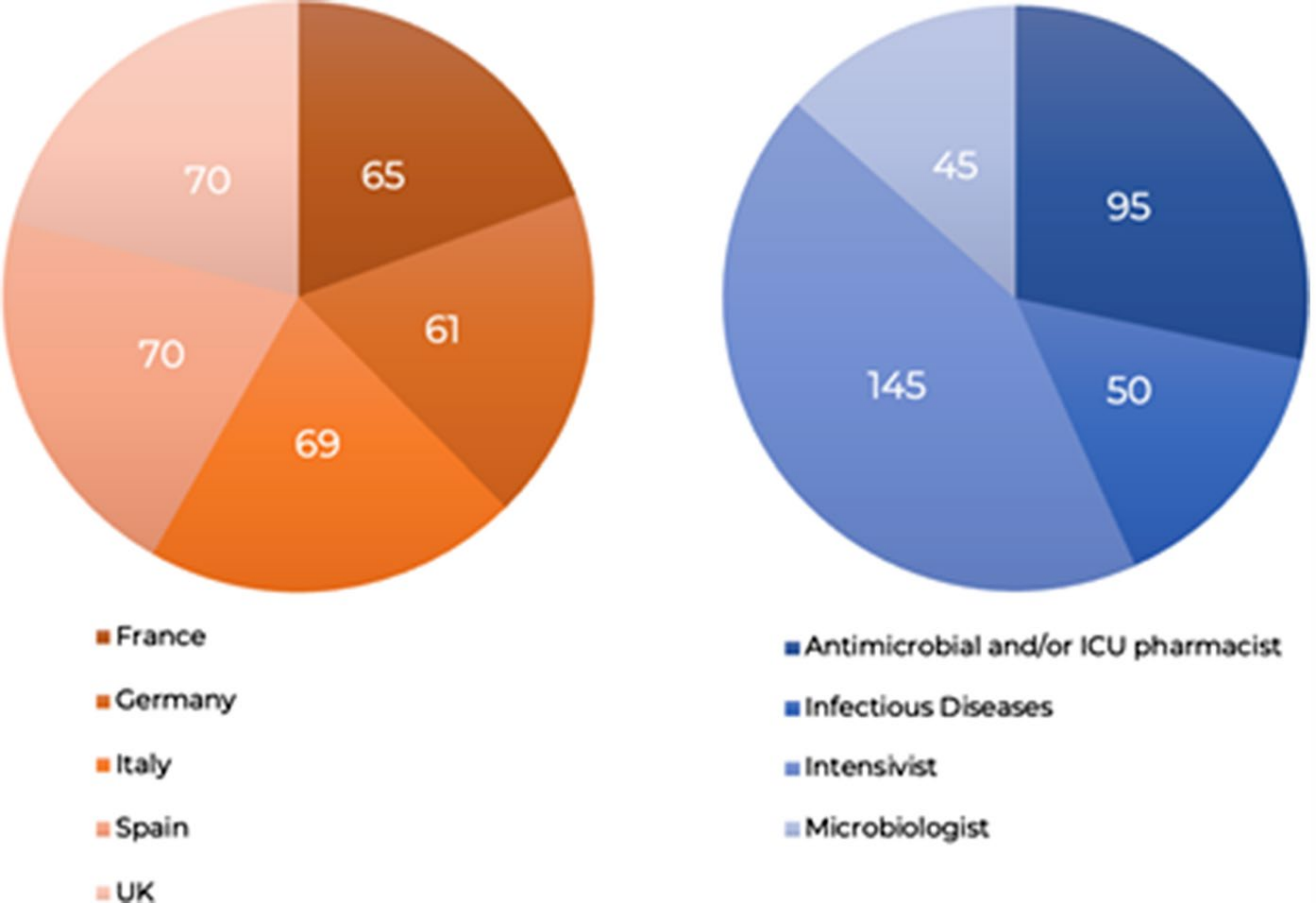
**A** number of responses received per country, **B** respondent reported specialities

During the five-month period to collect responses, a total of 335 responses were received. The number of responses received per country are displayed in Fig. 1A, responses according to medical specialisation in Fig. 1B, with the majority of responses received from intensivists (n = 145), followed by antimicrobial and/or ICU pharmacists (n = 95). From these responses, 29/44 (66%) statements attained very high agreement (≥ 90%), 11/44 (25%) high agreement (< 90% and ≥ 75%). The remaining 4/44 (9%) did not meet the established threshold for consensus (< 75%). The full set of statements and corresponding levels of agreement are shown in Table 1.

After review of the results, and given the high level of consensus achieved for 40/44 statements (91%) and that the stopping criteria had been met, the panel agreed that a single round of testing was sufficient for the purposes of this study. The panel agreed that statements which did not achieve consensus in this round should be reported as such and that for the purposes of this study should not be amended and resubmitted to responders.

**Fig. 2:**
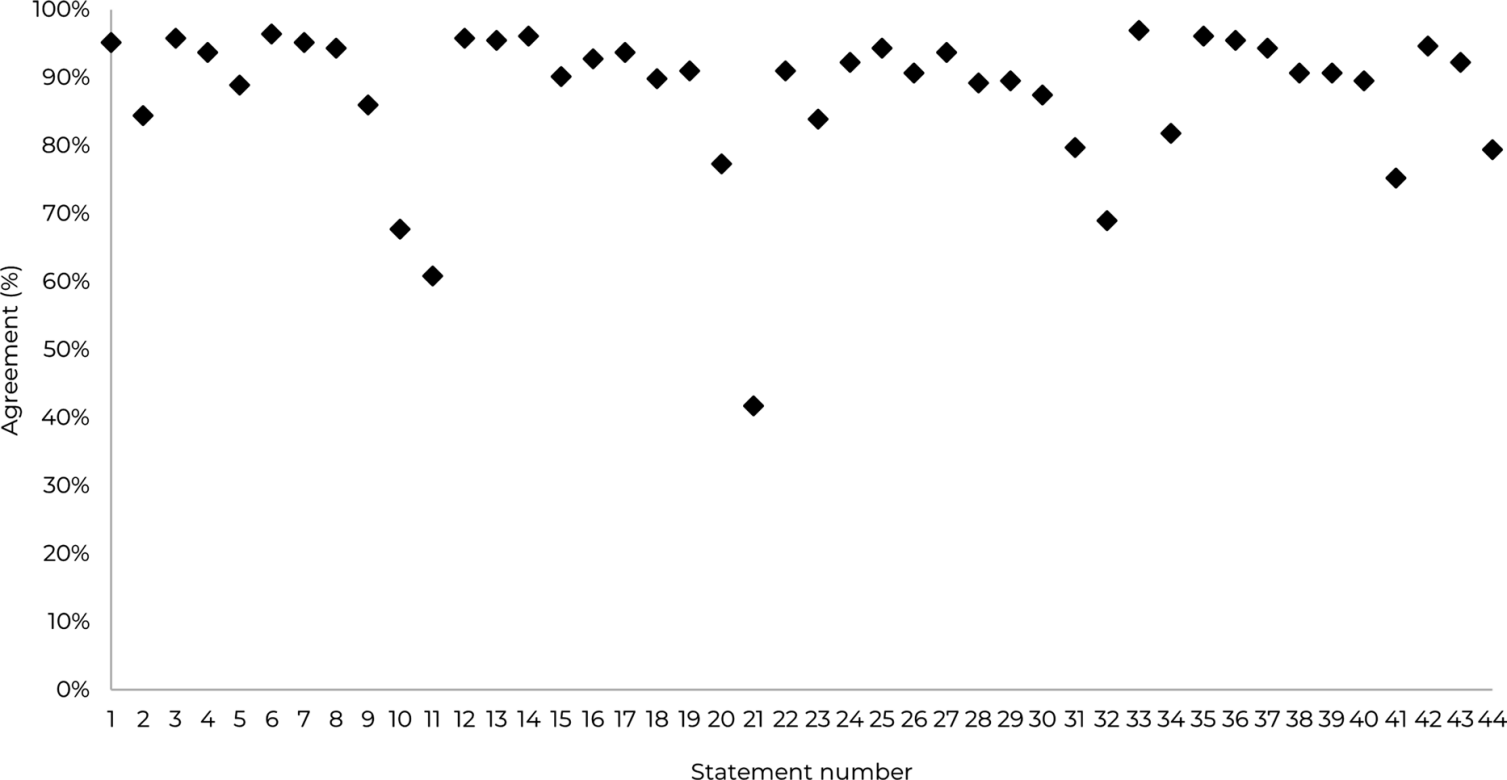
Combined consensus scores from 335 responses. *Note* the green line represents the threshold for consensus (75%) and the blue line represents the threshold for very high agreement (90%)

**Fig. 3:**
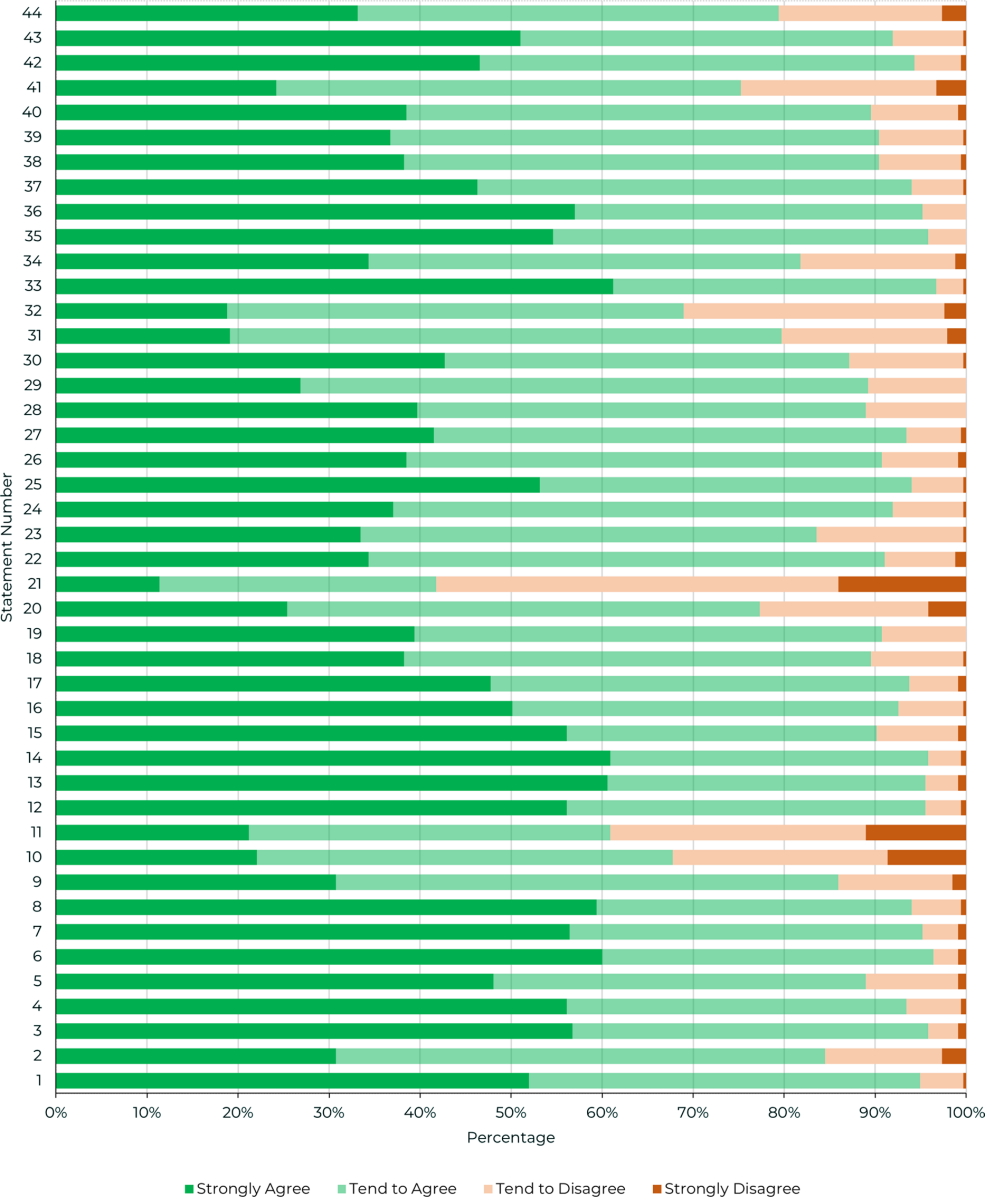
Consensus score distribution across the four-point Likert scale provided to respondents

The results of the survey show a high degree of agreement across the consensus topics (Figs. 2, 3), with respondents showing strong consensus around the patient characteristics of IFI cases (Statements 1–9). HCPs also strongly agreed with the need to initiate treatment as quickly as possible where IFI is suspected, (Statements 17–30), and highlighted the need for greater evidence requirements in areas like incidence of IFIs and antifungal prophylaxis (Statements 37–44).

Upon analysis of results, clear trends emerged. When examining response by country (Fig. S1), the UK had the highest average level of agreement to the statements compared to France and Germany, which tended to be lower.

Analysis of response by speciality (Fig. S2) revealed some clear differences in agreement level, for example, response to Statement 21 (voriconazole is not a suitable treatment option for ICU patients) amongst infectious disease specialists and intensivists showed the lowest level of agreement (32% and 34% respectively) whereas microbiologists showed the highest agreement (69%).

Most respondents agreed that a BDG or GM test would take longer than 3 days in their institution (Statement 10, 68%; Statement 11, 61%). Analysis by country indicates that the UK, Italy, and France experience delays greater than 3 days for turnaround of BDG test and serum GM (BDG: 81%, 75% and 80% respectively. GM: 79%, 72% and 72% respectively).

To improve the timing and accuracy of IFI diagnosis (Statements 12, 13, and 14, all 96%), respondents strongly agreed that access to (i) serum BDG test results, (ii) serum or BAL GM test results, and (iii) PJP polymerase chain reaction results should be available in all ICU facilities within around 48 h. Furthermore, there was also strong agreement that fungal culture, species identification, and susceptibility testing should all be available in-house at every hospital. 77% of respondents agreed that a serum BDG antigen test should be taken before initiation of any antifungal treatment for patients with suspected *Candida* infection (Statement 20).

Strong agreement was observed that the choice of treatment for an IFI in the ICU should be based on the fungal pathogen, susceptibility testing, pharmacokinetics, co-morbidities, and the site of infection (Statements 15, 25 and 35, 90%,94% and 96%, respectively).

## Discussion

In this study, an expert panel developed 44 statements across 6 key domains concerning the diagnosis and management of IFI in the ICU. These statements were then evaluated by an online survey with 335 respondents to identify unmet needs and key recommendations to improve IFI care.

Regarding identification of patients at risk of IFIs, data show a wide variation in incidence across geographies [28], therefore local incidence rates should be available for Physician use in determining individual risk. Individual patient characteristics must also be considered, as risk groups differ between IA (patients with COPD, patients who are immunocompromised, patients with viral pneumonia, liver cirrhosis, or autoimmune diseases) and invasive *Candida* (IC) infections (any patient with disruption of the skin and gastro-intestinal barrier, and those receiving treatments that change the composition of the gut microbiome). [16, 28–30]

Early reliable diagnosis of IFI is of great importance, particularly for IA due to the associated very high mortality rates following angio-invasion despite appropriate antifungal treatment [31]. The ideal sample for diagnosing IA in the ICU is a bronchoalveolar lavage (BAL) for galactomannan (GM) and culture. Additionally, treatment delay in IC infections is a major factor predicting mortality [32, 33], as echoed in the 90% agreement of respondents in this study.

A major obstacle to early diagnosis is the long turnaround time to receive results from mycological tests, and a turnaround time of longer than 3 days for BDG and GM tests was reported by the majority of respondents in this study, suggesting a clear and measurable potential metric for improvement. For the UK, turnaround time for has previously been reported for BDG (> 48 h in 87% of cases) and GM (> 48 h in 86% of cases) [34]. These data were driven by the fact that few laboratories had access to local testing for BDG (3/63, 5%) or GM (13/63, 21%), and most send samples out to reference laboratories [35]. The turnaround time for these tests may be even longer in the more resource-limited countries in Europe, as shown recently by the European Confederation of Medical Mycology (ECMM) laboratory capacities survey. [1]

These results suggest that where possible, all fungal testing should be conducted in-house to ensure a rapid turnaround time (< 48 h) and provide timely patient care in this setting where every hour counts. This recommendation could become a target for programmes such as the ECMM, International Society of Human and Animal Mycology (ISHAM), and the American Society for Microbiology (ASM) One World–One Guideline initiative [26, 36] and other initiatives. Another obstacle to early diagnosis is the lack of reliable diagnostic criteria for IA in patients who are non-neutropenic, as the established criteria for the neutropenic setting often cannot be applied to those patients who are non-neutropenic (Statement 9, 86%). Currently, efforts are underway to create improved classification criteria for the ICU setting. [37]

Early initiation of appropriate antifungal therapy is essential for a successful outcome of IFI [38], and this was recognised in the current study with strong agreement that empirical treatment should start immediately and prior to receiving the results from any diagnostic test if there is high clinical suspicion and, especially, high clinical severity.

Regarding the use of serum BDG testing prior to initiation of antifungal treatments, it is interesting to note that lower agreement levels to this statement were observed from respondents in Germany, Spain, and France (70%, 73%, and 69% respectively). These were considerably lower than responses from the UK (83%) and Italy (90%). This may be due to varying availability, turnaround time, and reimbursement of serum BDG testing. For example, guidance within the UK National Health Service recommends the use of serum BDG testing, but does not mandate it [39].

Evidence suggest that serum BDG has a use in guiding the cessation of empirical antifungal therapy via a negative result [40] because of the high negative predictive value (NPV) the test presents (of around 90–95%) [22]. Given that serum BDG tests become negative under appropriate antifungal therapy (and are therefore also used for treatment monitoring) [41] it is therefore important to obtain a sample for serum BDG testing before initiation of therapy, otherwise the NPV would decrease [42]. Combination of BDG with procalcitonin testing (negative PCT in the presence of candidemia) has shown promise, but needs further exploration, also due to the limited specific diagnostic performance of PCT in this setting[43, 44]. Receiving serum BDG results within 48 h, would allow for early discontinuation of antifungals, and prevent prolongation of unneeded antifungal treatment. In contrast, serum BDG may be less reliable as a marker driving the initiation of antifungal therapy in suspected *Candida* infection with positive test results. Data from a recent randomised controlled trial examining the use of antifungal therapy in ICU patients with sepsis at a high risk of *Candida* infection determined that, in this select group of at-risk patients, serum BDG-guided initiation of antifungal therapy did not improve 28-day mortality and may be associated with overtreatment (i.e., treating patients with antifungals without IC infection) [45].

Therefore, with the support shown by the respondents to the statements (particularly Statement 31, 80%), serum BDG should be taken at treatment initiation and the results used thereafter to help guide the discontinuation of empirical antifungal therapy for IC when there is no further evidence of current infection. Despite being supported by a strong recommendation (low quality evidence) by the European Society of Intensive Care Medicine-European Society of Clinical Microbiology and Infectious Diseases *Candida* infection task-force [46], there was a lack of consensus observed for Statement 32, “*In treatment of suspected (and not proven) Candida infection without a clear clinical focus, if no improvement in clinical situation is seen within 4–5 days, then treatment can be stopped”* (69%). This suggests a view amongst respondents that clinical findings alone are not sufficient for the discontinuation of treatment and further outlines the need for more BDG testing to support decisions on IC treatment discontinuation. The use of biomarkers can support the decision to stop antifungal treatment.

Results suggest that the choice of initial treatment the choice of treatment for an IFI in the ICU should be based on the fungal pathogen, susceptibility testing, pharmacokinetics, co-morbidities, and the site of infection. This highlights the importance of knowing the local epidemiology of *Candida* species and susceptibility to increase the likelihood of appropriate empiric antifungal treatment, while waiting for the microbiological results of susceptibility testing. Drug-drug interactions should also be considered (Statement 34, 82%) as low drug levels are one of the potential effects of drug interactions, hospitals should therefore consider their ability to perform therapeutic drug monitoring (TDM) when selecting an antifungal treatment. This seems particularly relevant for voriconazole treatment where TDM is strongly recommended in guidelines [47–49], but not always available in institutions across Europe [1]. Furthermore, a recent ECMM survey of IFI diagnostic capacity in Europe showed that availability of TDM was closely related to the gross domestic product of the country in which the institution was located [1].

In cases where culture/specific diagnostics are not possible, a broad-spectrum antifungal treatment should be selected. In cases of suspected mould infections, these broad-spectrum agents would include liposomal amphotericin B, isavuconazole, and posaconazole with activity not only against *Aspergillus* (for which voriconazole would be a viable option [8]) but a wide range of non-*Aspergillus* moulds [26]. For *Candida* infections, echinocandins would be the initial treatment of choice. [50] It was recognised that azole resistance in *Aspergillus* [51] and other fungal pathogens [18] is an emerging problem that requires future actions. HCPs agreed that in case of local azole resistance rates of > 10%, alternative antifungal treatment should be considered empirically.

In terms of special populations in the ICU, patients on extracorporeal membrane oxygenation (ECMO) were identified by respondents as a population with great unmet needs and who tend to achieve generally lower serum levels of antifungals [52].Therefore, either higher dosages or TDM with rapid dosage adjustments may be required. However, it was also acknowledged that data on antifungal treatment of patients on ECMO are limited, and further studies are needed. Renal replacement therapy in individuals with renal impairment should also be considered when selecting antifungal treatment (Statement 36, 96%).

Respondents agreed that both fungicidal mode of action in abdominal infections, and drug-drug interactions are important factors to consider when selecting a treatment. This may have contributed to the 42% agreement that voriconazole is not a suitable treatment option in ICU patients (Statement 21). However, if voriconazole is used for treatment, TDM is essential with results available within 48 h after achieving steady state (Statement 22). If TDM of voriconazole shows a failure to achieve therapeutic levels after reaching steady state, either a dose escalation or a change to another antifungal is the most appropriate strategy (Statement 24).

Our study also outlines important areas for education and evidence building. There are clear differences of opinion over the role of voriconazole treatment and antifungal prophylaxis between respondent specialities and country (Fig. S2, Statements 21 and 41). This suggests that there is an opportunity to raise awareness of what constitutes optimal treatment modalities amongst all roles. The general lack of confidence reported amongst HCPs regarding the use of antifungal agents and interpreting diagnostic results, supporting a need for greater education of these often rare and orphan diseases. Proactive formal training, with a rigorous education package around IFI, could help improve clinical confidence. This training should include available treatment methods to combat infections and provide the necessary support to implement antifungal use.

### Identified Areas of IFI Management in Need of Improvement

Based on the results obtained over the course of this study and the discussion held by the expert panel to review the findings, the authors have identified the following areas requiring attention and the below measures as a way forward as to how to address these remaining IFI challenges in the ICU (Table 2).

**Table 2:** Expert panel proposed measures to address challenges identified from the ICU consensus survey

No	Measure
*Clinical standards / areas for improvement*	
1	All testing should be completed in-house where available to reduce turnaround times and increase efficiency of care
2	Access to testing for IFIs should be available at all ICU centres, with provisions made to supply point-of-care and single-sample tests to smaller centres or where laboratories are centralised and off-site
3	Mycological test results should be available within 48 h to aid diagnosis, appropriate treatment initiation and discontinuation
4	Serum beta-D-glucan tests should be used prior to the initiation of treatment with antifungal agents in patients with suspected *Candida* infections to guide the early discontinuation of treatment where appropriate
*Educational priorities*	
5	Education around appropriate diagnostic tests and interpretation of the results for different types of IFI (and particularly that of invasive aspergillosis) should be increased as well as education on the appropriate use of different antifungal treatments to improve patient outcomes
6	Physicians should be aware of the incidence of different IFIs and their associated rate of azole resistance, especially for Aspergillus and non-albicans Candida species in their unit. Centralised databases should be utilized to improve the standards of care within their institution for patients with high risk factors for IFI
7	To improve the standards of care for patients affected by IFI, and support clinicians in the management of IFIs, a rigorous education package should be provided around the utilisation of antifungal agents whilst providing the necessary support to implement their use as swiftly as possible when IFI is suspected
*Further research priorities*	
8	A greater body of evidence around the benefit of prophylaxis for IFIs in the ICU should be developed

This study has a number of strengths. The main strength of this study compared to expert statement publications is the validation of the consensus statements by a multidisciplinary cohort of healthcare professionals involved in the ICU management of IFIs in five different European countries. There was good representation across all countries included within the study with over 300 respondents, lending greater weight to what ICU healthcare professionals actually view as current status of care, optimal care and subsequent challenges faced in their clinical practice. The large study population provides further weight to the findings, especially given the distribution of results across the ICU roles tested.

The study had some limitations. Firstly, the wording of some statements may have been ambiguous and therefore have impacted the agreement displayed. Statements were developed by the expert panel and then ratified by each member individually before being used to develop the survey to reduce any potential bias that may have emerged. Delphi methodology is intended to test opinions. As such, the statements were drafted by authors with clinical and scientific expertise in the field and written as assertions. However, they could be perceived by some as encouraging agreement, although a full scale of opinion was invited from all responders.

Secondly, responses were sought using an incentivised methodology through a standard market research approach, which may have introduced participant bias. Respondents may have felt pressured to align their answers with the perceived objectives of the research group.

Thirdly, respondents’ expertise in IFI management was likely heterogeneous. However, all respondents were screened to confirm involvement in IFI diagnosis and management and had to have a predefined number of years of experience in their roles, so responses were valid in reflecting the current situation in centres across Europe.

Considering these factors, all results were analysed by the project facilitators for potential problems. The full data set was also presented to the expert panel for ratification and to ensure that no bias was present or inadvertently introduced.

Finally, although externally validated, the proposed statements and measures have been developed through modified Delphi consensus approach and as such are expert opinions of the panel and respondents and as such are not evidence-based recommendations.

## Conclusions

Based on the results obtained over the course of this study and the discussion the expert panel held to review the findings, the authors identified a number of areas where measures could be taken to address the current state of IFI management. These measures could serve as a potential path on how to improve IFI diagnosis and management, thereby helping to address the remaining IFI challenges in the ICU. These consensus statements and measures may also guide areas for further research to optimise the management of IFI in the ICU.

### Supplementary Information

Below is the link to the electronic supplementary material.

Supplementary file1 (DOCX 23 kb)

Supplementary file2 (DOCX 21 kb)

## Data Availability

The datasets used and/or analysed during the current study are available from the corresponding author on reasonable request.

## Abbreviations

ASM: American society for microbiology
BAL: Bronchoalveolar lavage
BDG: Beta-D-Glucan
CAPA: COVID-19 Associated pulmonary aspergillosis
CAM: COVID-19 Associated pulmonary mucormycosis
COPD: Chronic obstructive pulmonary disease
ECMM: European confederation of medical mycology
ECMO: Extracorporeal membrane oxygenation
GM: Galactomannan
HCPs: Healthcare professionals
IA: Invasive aspergillosis
IC: Invasive *candida*
ICU: Intensive care unit(s)
IFI: Invasive fungal infection(s)
ISHAM: International society of human and animal mycology
NPV: Negative predictive value
PJP: *Pneumocystis* *jirovecii* Pneumonia
TDM: Therapeutic drug monitoring
