# Supplementary material for: Exploring European Consensus About the Remaining Treatment Challenges and Subsequent Opportunities to Improve the Management of Invasive Fungal Infection (IFI) in the Intensive Care Unit
Source: Mycopathologia. 2024 May 5;189(3):41. doi: 10.1007/s11046-024-00852-3 (PMC11070387; doi:10.1007/s11046-024-00852-3)
Supplement: Supplementary file 2 — Supplementary file2 (DOCX 21 kb) [file 11046_2024_852_MOESM2_ESM.docx]

**Fig. S2** Consensus scores displayed by respondent reported specialty.

Note: the green line represents the threshold for consensus (75%).
